# Effect of oxygen on the germination and culturability of *Bacillus atrophaeus* spores

**DOI:** 10.1007/s10123-021-00229-2

**Published:** 2022-01-07

**Authors:** Wen Jie Wu, Jinhui Chang

**Affiliations:** 1 Department of Radiation Physics, The Cancer Hospital of the University of Chinese Academy of Sciences (Zhejiang Cancer Hospital), Institute of Basic Medicine and Cancer (IBMC), Chinese Academy of Sciences, Hangzhou, 310022 Zhejiang China; 2 Zhejiang Key Laboratory of Radiation Oncology, Hangzhou, 310022 Zhejiang China; 3 Department of Applied Biology and Chemical Technology, The Hong Kong Polytechnic University, Hong Kong, China

**Keywords:** *Bacillus atrophaeus*, Spore germination, Culturability, Oxygen

## Abstract

The effect of oxygen on the germination and culturability of aerobic *Bacillus atrophaeus* spores was investigated in this study. Under oxic or anoxic conditions, various nutritional and non-nutritional germinants were utilized to induce germination. Tb^3+^-dipicolinic acid fluorescence assay and phase-contrast microscopy were used to track the germination process. The final germination level, germination half time, and germination speed were used to define germination kinetics. Colony-forming unit enumeration was used to assess the culturability of germinated spores germinated with or without oxygen. The results show that in the absence of oxygen, the final germination level was unaffected, germination half time decreased by up to 35.0%, germination speed increased by up to 27.4%, and culturability decreased by up to 95.1%. It is suggested that oxygen affects some germinant receptor-dependent germination pathways, implying that biomolecules engaged in these pathways may be oxygen-sensitive. Furthermore, spores that have completed the germination process in either anoxic or oxic conditions may have different culturability. This research contributed to a better understanding of the fundamental mechanism of germination.

## Introduction

One of the most astonishing aspects of spore-forming bacteria is their capacity to survive in harsh environment, partly due to their unique two-life-cycle system. Vegetative growth and binary fission can be observed when the environment is conductive to metabolism. In the presence of external stresses such as nutrient deprivation, vegetative cells enter the sporulation cycle, during which spore-forming bacteria form spores to protect themselves from being inactivated. Spores are dormant structures produced by gram-positive bacteria, primarily *Bacillus* and *Clostridium* species. Spores are highly resistant to heat, chemical oxidation, UV irradiation, and toxic substances due to their low-permeability inner membrane (IM), extremely dehydrated spore core, large depot of Ca^2+^-dipicolinic acid (Ca-DPA), and α/β-type small, acid-soluble proteins (SASPs) bound DNA. Spores can keep dormant for thousands of years with no detectable metabolism (Christie et al. 2020). Dormant spores, on the other hand, continue to sense their surroundings. When favorable conditions arise, they germinate quickly, followed by outgrowth and finally return to vegetative forms. As a result, incomplete eradication of spores is connected with a risk of producing major public health concerns such as food contamination (Brown 2000; Burns et al. 2010), nosocomial infections (Wilcox et al. 2000), and even a threat to national security (McCarthy 2001).

Germination, as a vital step in the process through which a spore returns to a vegetative cell, has piqued the curiosity of many scientists and has resulted in countless fundamental and practical studies since the mid-twentieth century. Germination can be triggered by certain chemicals termed as germinants, which include both nutrients and non-nutrients. Nutrient germinants can be simple amino acid (e.g., L-alanine) or mixed compounds (e.g., L-asparagine, glucose, fructose, and K^+^ (AGFK)). Non-nutrient germinants include Ca^2+^-dipicolinic acid (Ca-DPA) (Paidhungat et al. 2001), cationic surfactants (B. Setlow et al. 2003), and so on. In addition to germinants, germination can also be triggered by high pressure (Paidhungat et al. 2002) or initiate spontaneously (Powell et al. 1955). Germination process is also influenced by many other factors. Pre-heat shock, for example, may activate germinant receptors (GRs) (Luu et al. 2015); inoculum size may impact germination via quorum sensing (Caipo et al. 2002); and sporulation condition may affect GR level (Hornstra et al. 2006; Ramirez-Peralta et al. 2012a, 2012b).

Germination, considered as a biophysical degradative process, has been extensively investigated from the molecular to the cellular level. However, the importance of oxygen in germination has been debated for more than a half century. Roth et al. discovered that some aerobic *Bacillus* spores may germinate in anoxic circumstances (Roth et al. 1956). Hagen and colleagues reported that the oxygen level affected the moisture requirement of *Bacillus atrophaeus* (*B. atrophaeus*) and *Bacillus subtilis* (*B. subtilis*) spore germination. When the moisture levels were in a specified range, they were able to germinate (but not outgrow) in anoxic conditions (Hagen et al. 1967). Wynne et al. discovered that anaerobic *Clostridium perfringens* and *Clostridium chauvoei* spores could germinate in an aerobic environment even in the presence of streptomycin (Wynne et al. 1952). These studies demonstrated that an oxic/anoxic environment is not required for the germination of aerobic/anaerobic spores, implying that germination resembles to be a purely biophysical process. Respiration during germination, on the other hand, has been reported in several early literatures. Levinson et al. discovered that oxygen was consumed during germination, and the respiration rate of *B. megaterium* spores was influenced by germination circumstances such as pre-incubation, germinant, heat activation, buffer, and pH (Levinson et al. 1956). Setlow and Kornberg’s research supported Levinson’s findings. Setlow et al*.* split the ATP level against time during germination into three stages and discovered that ATP production was anaerobic in stage I and aerobic in stage III (Setlow et al. 1970a). Fujioka and Frank reported only 28% germination ratio of putrefactive anaerobe 3679 (later taxonomically termed as *Clostridium sporogenes*) spores in alanine-deficient medium under oxic conditions, but the ratio reached 100% in anoxic conditions (Fujioka et al. 1966). All of these opposing opinions underscore the importance of determining the role of oxygen in germination. However, until today, quantifiable data connecting oxygen to spore germinability and culturability were few.

To investigate the effect of oxygen on aerobic spore germination, special consideration is needed in selecting *Bacillus* species. The species should be obligate aerobic so that spores germinated in anoxic conditions cannot enter the phases of outgrowth and vegetative metabolism without additional oxygen supply, which facilitates the germination study. Meanwhile, non-pathogen surrogate is preferred due to laboratory safety concerns. Taking these factors into account, obligate aerobic *B. atrophaeus* was chosen as the model species.

In this work, the influence of oxygen on the germination and culturability of *B. atrophaeus* spores was examined using a variety of methodologies. Specifically, Ca-DPA release was tracked by Tb-DPA fluorescence assay, refractility change was observed by phase-contrast microscopy, and culturability was determined by enumerating the number of colony-forming unit (CFU) formed from spores that germinated with or without oxygen. The mechanisms that may produce these disparities were explored.

## Materials and methods

Unless stated, all chemicals were purchased from Sigma-Aldrich (St. Louis, MO, USA) and used without further purification, and all experiments were carried out in triplicate at room temperature (25 ± 2 °C).

### Production of spore

*B. atrophaeus* (ATCC 9372) spore strip was incubated in 30 g/L tryptic soy broth (TSB) as a general growth medium at 37 °C for 18 h. Exponentially growing vegetative cells were inoculated into a sporulation medium, which consisted of 1.6% nutrient broth, 1.5% agar, 0.2% KCl, 0.05% MgSO_4_, 1 mM Ca(NO_3_)_2_·4H_2_O, 100 µM MnCl_2_·4H_2_O, 1 µM FeSO_4_·7H_2_O, and 0.1% glucose. All components were sterilized by filtering (use 0.2-µm filter) or autoclaving. The cells were incubated at 37 °C under ambient conditions for 4 days. Following an overnight lysozyme (500 µg/L, 0.2–µm-filter-sterilized) digestion at 37 °C, spores were purified by 10 times of centrifugation at 11,180 × g at 4 °C and washing until a 99.9% purity was reached, as verified using a phase-contrast microscope (AZ100, Nikon). The concentration of spore suspension was then determined using a hemocytometer (Petroff-Hausser, Horsham, PA, USA). Stocks of spore suspension were stored at 4 °C in the dark (Chang et al. 2021).

### Preparation of media and solutions

L-alanine germinant solution contained 50 mM L-alanine. AGFK germinant solution contained 50 mM L-asparagine, glucose, fructose, and potassium chloride. Both were buffered in 50 mM Tris–HCl at pH = 7.6. TSB germinant medium contained 30 g/L TSB. Ca-DPA germinant medium was made from a 1:1 mixture of 100 mM CaCl_2_ and 100 mM DPA. The pH of DPA was adjusted to 7.5 by 10% Trizma® base solution before mixing (Peng et al. 2009). TSA culture medium comprised 15 g/L agar and 30 g/L TSB. An extra 100 µM TbCl_3_ was utilized as sensing molecules in Tb-DPA fluorescence assay. All media/solutions were prepared in deionized (DI) water from a Direct-Q Ultrapure Water Systems (Merck Millipore, Billerica, MA, USA) and autoclaved or 0.2-µm-filter-sterilized prior to use. All anoxic experiments were carried out in an anaerobic chamber (Coy Laboratory Products, Grass Lake, MI, USA) using a platinum–palladium catalyst in an environment of 3% hydrogen, 97% nitrogen, and < 20 ppm oxygen. In this study, anoxic media/solutions were defined as having dissolved oxygen (DO) levels of less than 0.1 mg/L. Anoxic DI water was produced by removing the DO from regular DI water in two steps. To summarize, DI water was autoclaved at 121 °C for 60 min before being moved to an anaerobic chamber to remove the DO predominantly. Remnant DO was further removed by placing DI water in the airlock of the chamber, vacuuming to 25 inHg and pumping in pure nitrogen (N_2_ ≥ 99.995%, O_2_ ≤ 10 vpm). This vacuuming-and-pumping process was repeated 10 times until DO < 0.1 mg/L was reached, as measured by a YSI 52 DO meter (YSI, Yellow Springs, OH, USA). All anoxic media/solutions were then prepared in the anoxic DI water and 0.2-µm-filter-sterilized prior to use.

### Germination study

#### Tb-DPA fluorescence assay

**Fig. 1:**
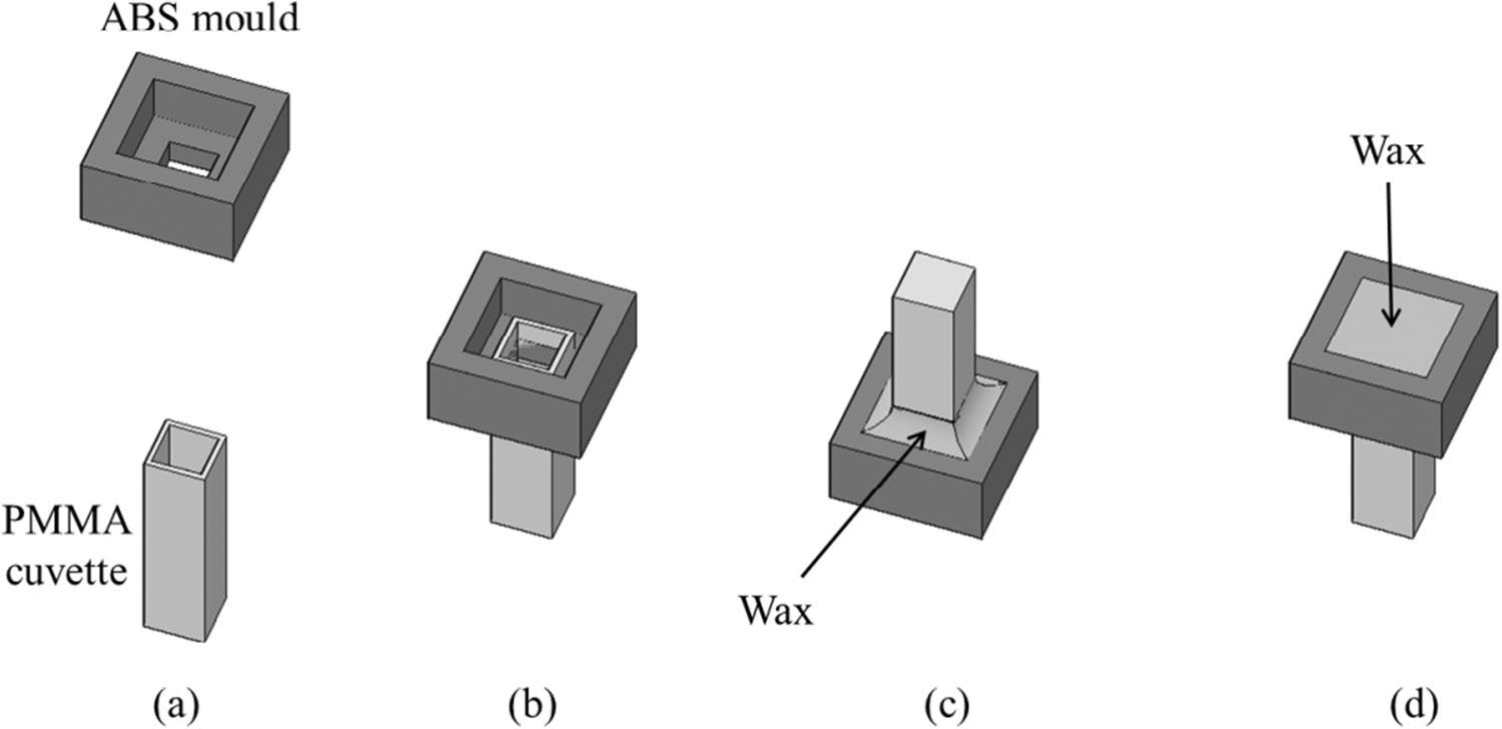
The schematic shows a cuvette insertion into an ABS mold (**a**, **b**), and the cuvette was further sealed underneath with wax (**c**). After adding the samples, the cuvette was then capped and sealed with wax to provide the anaerobic environment (**d**). This procedure was done in the anaerobic chamber

Tb-DPA fluorescence assay was used to monitor the DPA release during germination of bulk spore suspension. In the anaerobic experiment, germination medium was prepared in a poly(methyl methacrylate) (PMMA) cuvette (Dynalon, Rochester, NY, USA) to a final volume of 2 mL with spore (2 × 10^7^ cells/mL), TbCl_3_, and germinant (L-alanine or AGFK). Cuvette was inserted into an acrylonitrile butadiene styrene (ABS) mold and sealed with wax (Paraplast Plus, Sigma-Aldrich, USA) (Fig. 1). Sample was taken out from anaerobic chamber, and its emission spectra (λ_ex_ = 278 nm, λ_em_ = 450–560 nm) and excitation spectra (λ_ex_ = 250–360 nm, λ_em_ = 545 nm) were measured against time using a fluorometer (Fluorolog-3, Horiba Jobin Yvon, Edison, NJ, USA). The fluorescent intensity was denoted as ${I}_{\mathrm{anoxic}}(t)$. The protocols of oxic experiment were set up to be identical with that of the anoxic experiment, except that the entire process was carried out in ambient conditions using regular DI water. The fluorescent intensity was denoted as ${I}_{\mathrm{oxic}}(t)$.

Due to the collisional quenching effect caused by molecular oxygen, ${I}_{\mathrm{anoxic}}(t)$ was adjusted prior to further analysis. The adjustment curves were obtained from the fluorescent intensity against oxic/anoxic Tb-DPA solutions with various concentrations. In the Tb-DPA adjustment solutions, TbCl_3_ concentration was fixed at 100 µM, and DPA concentrations were 0, 2, 4, 6, and 8 µM. In our sporulation method, DPA content in each spore was determined to be 2.55 × 10^−16^ mol according to the method reported by Hindle (Hindle et al. 1999). Assuming all DPA was released upon germination, the final concentration of DPA in each sample cuvette was approximately 5.10 µM, which was within the range of DPA concentration in the adjustment solutions.

When Tb^3+^ concentration (${c}_{{\mathrm{Tb}}^{3+}}$) is higher than DPA concentration (${c}_{\mathrm{DPA}}$), the fluorescence (${I}_{\mathrm{oxic}}({c}_{\mathrm{DPA}})$) and DPA concentration have a linear relation (Rosen et al. 2001):

1 $${I}_{\mathrm{oxic}}({c}_{\mathrm{DPA}})={k}_{\mathrm{oxic}}{c}_{\mathrm{DPA}}$$

When DPA concentration and quencher (oxygen) concentration (${c}_{{\mathrm{O}}_{2}}$) is fixed, the ratio between fluorescence of anoxic Tb-DPA (${I}_{\mathrm{anoxic}}({c}_{\mathrm{DPA}})$) and oxic Tb-DPA (${I}_{\mathrm{oxic}}({c}_{\mathrm{DPA}})$) is constant:

2 $$\frac{{I}_{\mathrm{anoxic}}({c}_{\mathrm{DPA}})}{{I}_{\mathrm{oxic}}({c}_{\mathrm{DPA}})}=1+{k}_{q}{\tau }_{0}{c}_{{\mathrm{O}}_{2}}$$

where ${k}_{q}$ is the quencher rate coefficient and ${\tau }_{0}$ is the lifetime of fluorophore in the absence of quencher.

Therefore ${I}_{\mathrm{anoxic}}({c}_{\mathrm{DPA}})$ and ${c}_{\mathrm{DPA}}$ also have a linear relation:

3 $${I}_{\mathrm{anoxic}}({c}_{\mathrm{DPA}})=\left(1+{k}_{q}{\tau }_{0}{c}_{{\mathrm{O}}_{2}}\right){k}_{\mathrm{oxic}}{c}_{\mathrm{DPA}}={k}_{\mathrm{anoxic}}{c}_{\mathrm{DPA}}$$

Hence, both adjustment curves can be fitted by linear model. After obtaining ${k}_{\mathrm{oxic}}$ and ${k}_{\mathrm{anoxic}}$, the adjusted signal of ${I}_{\mathrm{anoxic}}(t)$ can be calculated:

4 $${I}_{\mathrm{anoxic}\_\mathrm{adj}}\left(t\right)=\frac{{k}_{\mathrm{oxic}}}{{k}_{\mathrm{anoxic}}}{I}_{\mathrm{anoxic}}\left(t\right)$$

In Tb-DPA fluorescence assay, Ca-DPA should not be used as germinant because the required concentration of exogenous Ca-DPA to trigger germination was around 40 mM (Riemann et al. 1961), which is much higher than the concentration of released DPA (~ 5.10 µM). TSB should not be used as germinant because the fluorescence will be severely disturbed by the light-yellow TSB medium.

#### Phase-contrast microscopy observation

Phase-contrast microscopy was used to observe the refractility change during germination of multiple individual spores. On a microscope slide (Marienfeld, Germany), a 2 µL droplet containing 2 × 10^8^ cells/mL spore suspension was gently dispersed and air-dried. Following that, a 5 µL droplet of germinant (L-alanine or AGFK or Ca-DPA or TSB) was placed over the dried spore pattern and sealed with a cover glass (thickness No. 1, Marienfeld, Germany). A thick coating of wax was applied on the cover glass borders to prevent water evaporation and gas exchange. For anoxic experiments, the slide was prepared in the anaerobic chamber using anoxic germinant before being taken out for microscopy examination. Images were captured with a CCD camera (SPOT Xplorer, Nikon, Japan). Each field of view contained 100 ± 20 spores. Germination ratios, denoted as ${R}_{\mathrm{anoxic}}(t)$ and ${R}_{\mathrm{oxic}}(t)$, were calculated as the number of phase dark body divided by the total number of spores in that view.

#### DO level measurement and estimation

DO in the anoxic medium is mainly derived from two sources: residual DO in anoxic water because of incomplete deoxidization and oxygen leakage cause by insufficient sealing after samples were taken out from the anaerobic chamber. The quantity of residual DO in anoxic DI water was continuously monitored using a DO meter throughout the deoxidize process until DO was less than 0.1 mg/L. The oxygen-sealing capacity of wax was determined by taking wax-sealed cuvettes holding anoxic DI water from the anaerobic chamber, placing them in ambient for 3 h, and then returning them to the anaerobic chamber. The DO level was then determined once more using the DO meter.

#### Culturability study

The relationship between oxygen availability during germination and the culturability of germinated spores was investigated using CFU enumeration. Oxic or anoxic germination was performed in a 500 µL volume of germinant medium containing spore (2 × 10^7^ cells/mL) and germinant (L-alanine or AGFK). After 2 h, spore suspension was diluted to 2 × 10^3^ cells/mL, and 100 µL suspension was transferred to TSA for spread plate. The plate was then incubated at 37 °C in an oxic environment for further outgrowth and vegetative growth of germinated spore. Meanwhile, two other sets of 100 µL spore suspension were directly inoculated on TSA at room temperature under oxic or anoxic conditions for 2 h. Both were then transferred to a 37 °C oxic condition for incubation, and CFU number was enumerated after 12 h. Ca-DPA cannot be utilized as germinant in this section because precipitate will be formed in the mixture of CaCl_2_ and DPA around 1–2 h after mixing, rendering serial dilution impossible.

Two further tests were performed as a supplement. The first is to measure the number of airborne bacteria in the anaerobic chamber using the settle plate method, as no biosafety cabinet was available during anoxic operation. To summarize, solid TSA was exposed directly to the anaerobic chamber atmosphere for 15 min before being moved to an oxic environment. After 12 h of incubation at 37 °C, the number of CFU was determined. The second is to confirm that the *B. atrophaeus* employed was obligate aerobic. Spores were incubated directly on TSA at 37 °C in an oxic or anoxic environment, followed by CFU enumeration after a suitable amount of time (see Results).

#### Germination kinetics modeling and statistical analysis

Germination data were fitted by the cumulative form of the Weibull distribution (Peleg et al. 2013):

5 $$P\left(t\right)={P}_{\mathrm{asym}}\left\{1-\mathrm{exp}\left[-{\left(\frac{t}{{t}_{c}}\right)}^{m}\right]\right\}$$

where ${P}_{\mathrm{asym}}$ was the asymptotic value and used as the final germination level, ${t}_{c}$ and $m$ were two factors that determine the steepness of $P\left(t\right)$. In this work, we define germination half time ${t}_{1/2}$ as the time when $P\left(t\right)$ reached the half value of ${P}_{\mathrm{asym}}$ and germination speed ${k}_{1/2}$ as the slop of tangent line of $P\left(t\right)$ at $t={t}_{1/2}$:

6 $$P\left({t}_{1/2}\right)=\frac{1}{2}{P}_{\mathrm{asym}}$$

7 $${k}_{1/2}={\left.\frac{dP\left(t\right)}{dt}\right|}_{t={t}_{1/2}}$$

From Eq. (5)–(7), we obtain

8 $${t}_{1/2}={t}_{\mathrm{c}}{\left(\mathrm{ln}2\right)}^\frac{1}{m}$$

9 $${k}_{1/2}=\frac{{P}_{\mathrm{asym}}m}{2{t}_{c}}{\left(\mathrm{ln}2\right)}^{\frac{m-1}{m}}$$

It can be derived that germination was promoted if ${t}_{1/2}$ was decreased or ${k}_{1/2}$ was increased (Peleg et al. 2013).

The values of ${t}_{1/2}$ and ${k}_{1/2}$ were obtained from curve fitting, and MATLAB® was used to calculate unpaired *t* test. Data points and error bars, respectively, stand for the average and standard deviation of triplicates.

## Results and discussion

### Assessment of dissolved oxygen level

At room temperature, the DO concentrations of oxic (unprocessed) and anoxic (deoxidized) DI water were 6.62 ± 0.32 mg/L and 0.07 ± 0.01 mg/L, respectively. The DO concentration of water collected from wax-sealed cuvettes that were left at ambient for 3 h was 0.08 ± 0.01 mg/L, indicating that the cuvette was oxygen-exclusive. Due to the fact that the spore stock (2 × 10^10^ cells/mL) was diluted 1000 times in the Tb-DPA fluorescence assay and culturability test, DO from the stock was less than 6.62 × 10^−3^ mg/L and can be ignored. In phase-contrast microscopy, the droplet of spore suspension was air-dried prior to adding anoxic germinant, leaving no DO from the stock.

### Effect of oxygen on spore germination

**Fig. 2:**
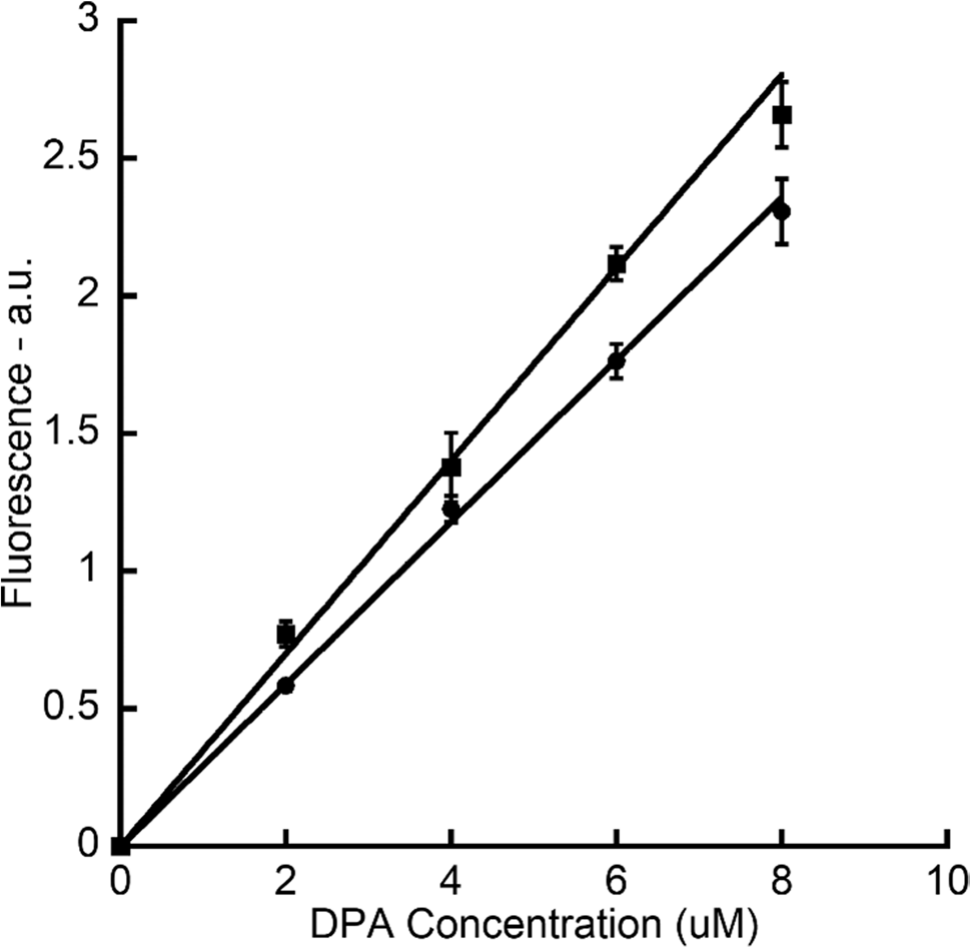
The correlation between fluorescent intensity (with arbitrary units [a.u.]) and oxic (●) or anoxic (■) Tb-DPA solutions with various concentrations

Although the fluorescence of anoxic and oxic Tb-DPA was different, both exhibited a linear relationship with the DPA concentration (Fig. 2). The linear coefficients were ${k}_{\mathrm{anoxic}}=3.51\times {10}^{7}$ and ${k}_{\mathrm{oxic}}=2.95\times {10}^{7}$ with arbitrary unit. Hence, ${I}_{\mathrm{anoxic}\_\mathrm{adj}}\left(t\right)=0.841 {I}_{\mathrm{anoxic}}\left(t\right)$.

**Fig. 3:**
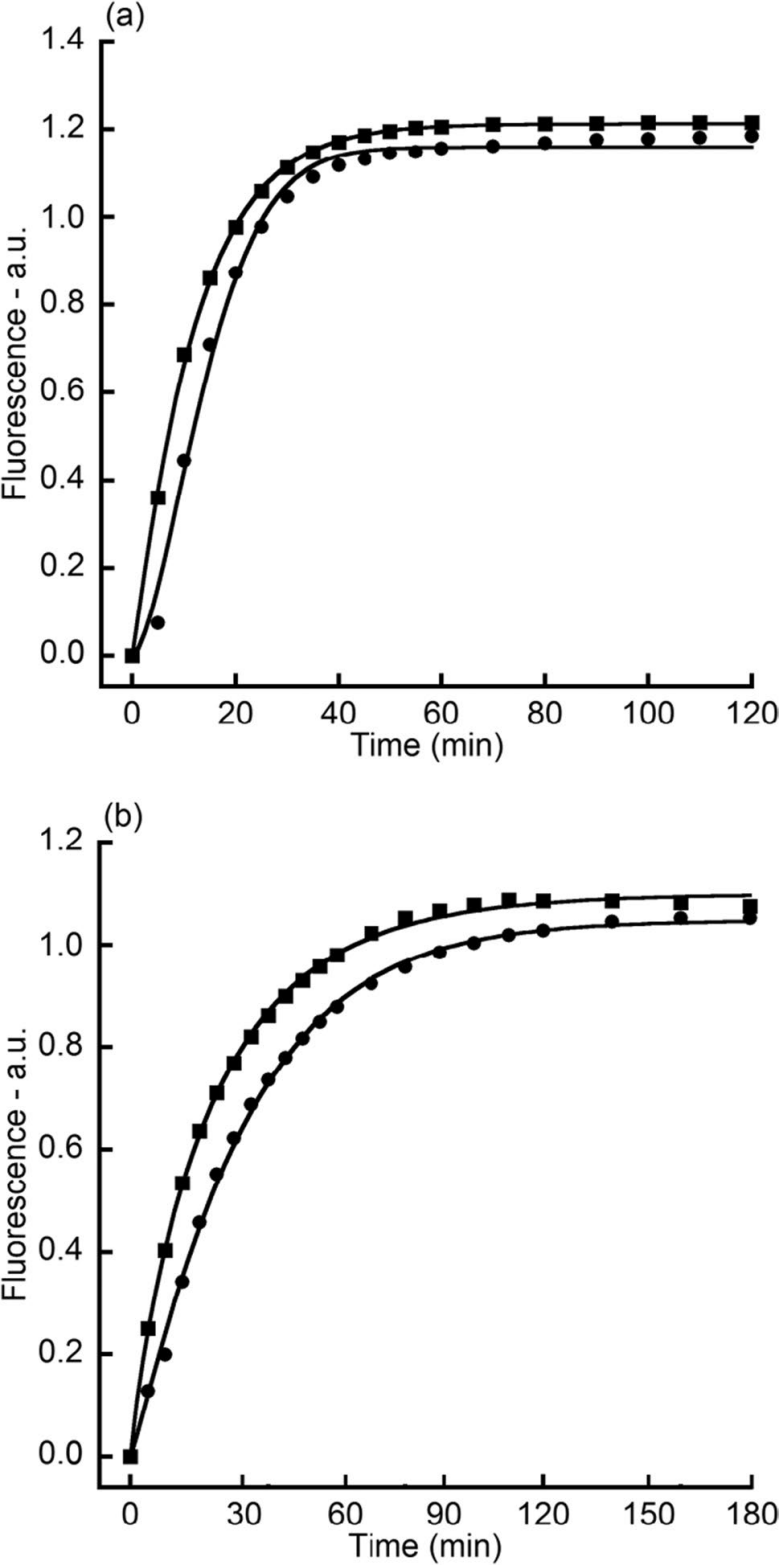
The graphs show the kinetics of germination of a spore population. *B. atrophaeus* spores were germinated with L-alanine (**a**) or AGFK (**b**) in oxic (●) or anoxic (■) conditions (see Materials and methods). Germination was monitored by Tb-DPA fluorescence assay. Data points the in anoxic group were adjusted according to Eq. (4). Data points in both groups were fitted using Eq. (5)

**Fig. 4:**
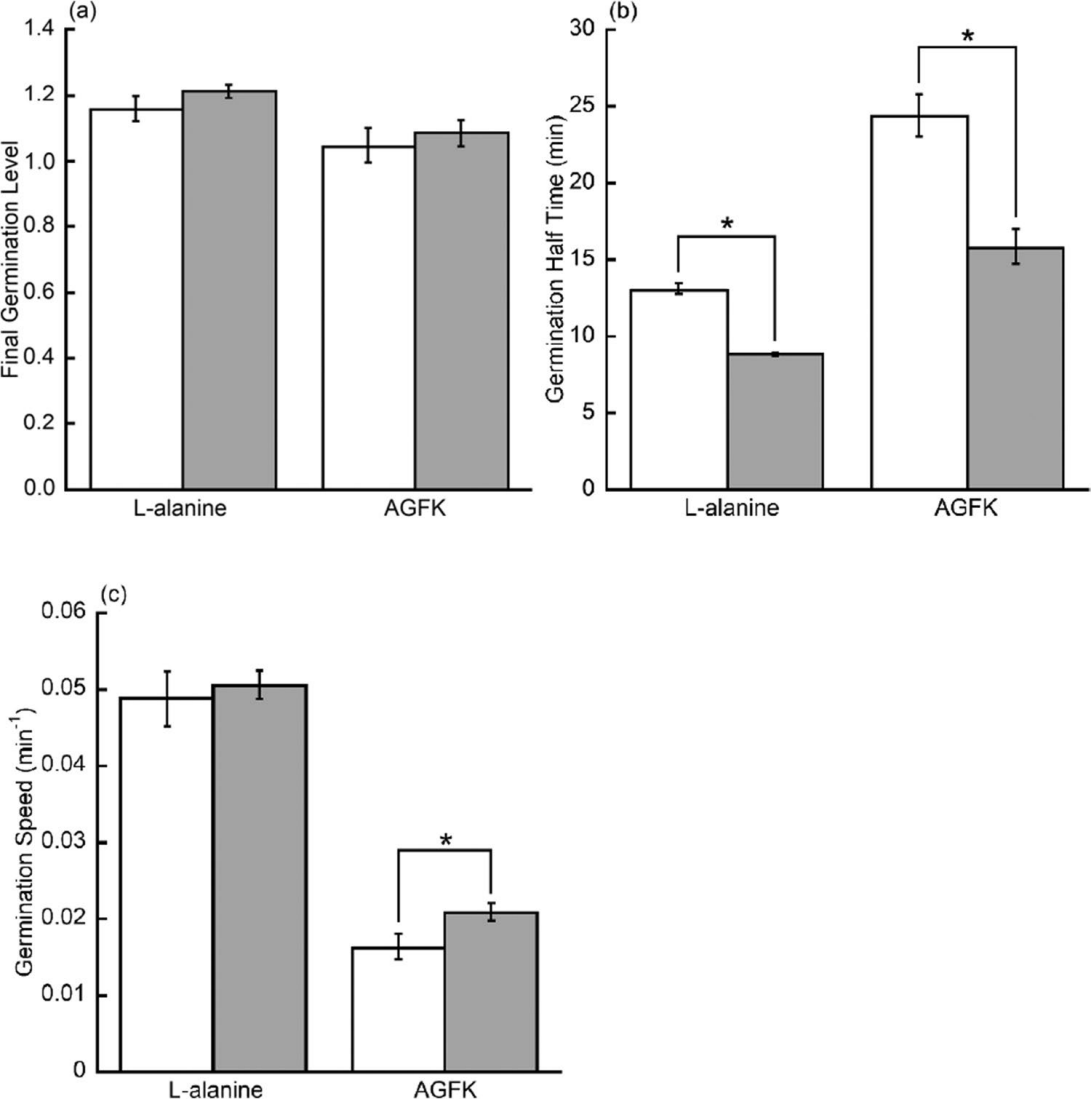
The graphs show the comparison of various germination parameters. *B. atrophaeus* spores were germinated in oxic (□) or anoxic (■) conditions (see Materials and methods). Germination was monitored by Tb-DPA fluorescence assay. Germination parameters were final germination level (**a**), germination half time (**b**), and germination speed (**c**). Significant differences (*) in germination half time (${t}_{1/2}$) were observed

The average value of triplicate ${I}_{\mathrm{anoxic}\_\mathrm{adj}}\left(t\right)$ and ${I}_{\mathrm{oxic}}\left(t\right)$ was fitted by Eq. (5) with ${R}^{2}>0.99$ for all 4 fittings (Fig. 3). Individual curve fitting of triplicate ${I}_{\mathrm{anoxic}\_\mathrm{adj}}\left(t\right)$ and ${I}_{\mathrm{oxic}}\left(t\right)$ yielded values for the final germination level, germination half time, and germination speed. These results (Fig. 4) indicated that in the absence of oxygen, germination half time decreased by 32.8% in L-alanine germination and by 35.0% in AGFK germination, while germination speed increased by 27.4% in AGFK germination. However, oxygen had no significant effect on the final germination level ($P>0.05$).

**Fig. 5:**
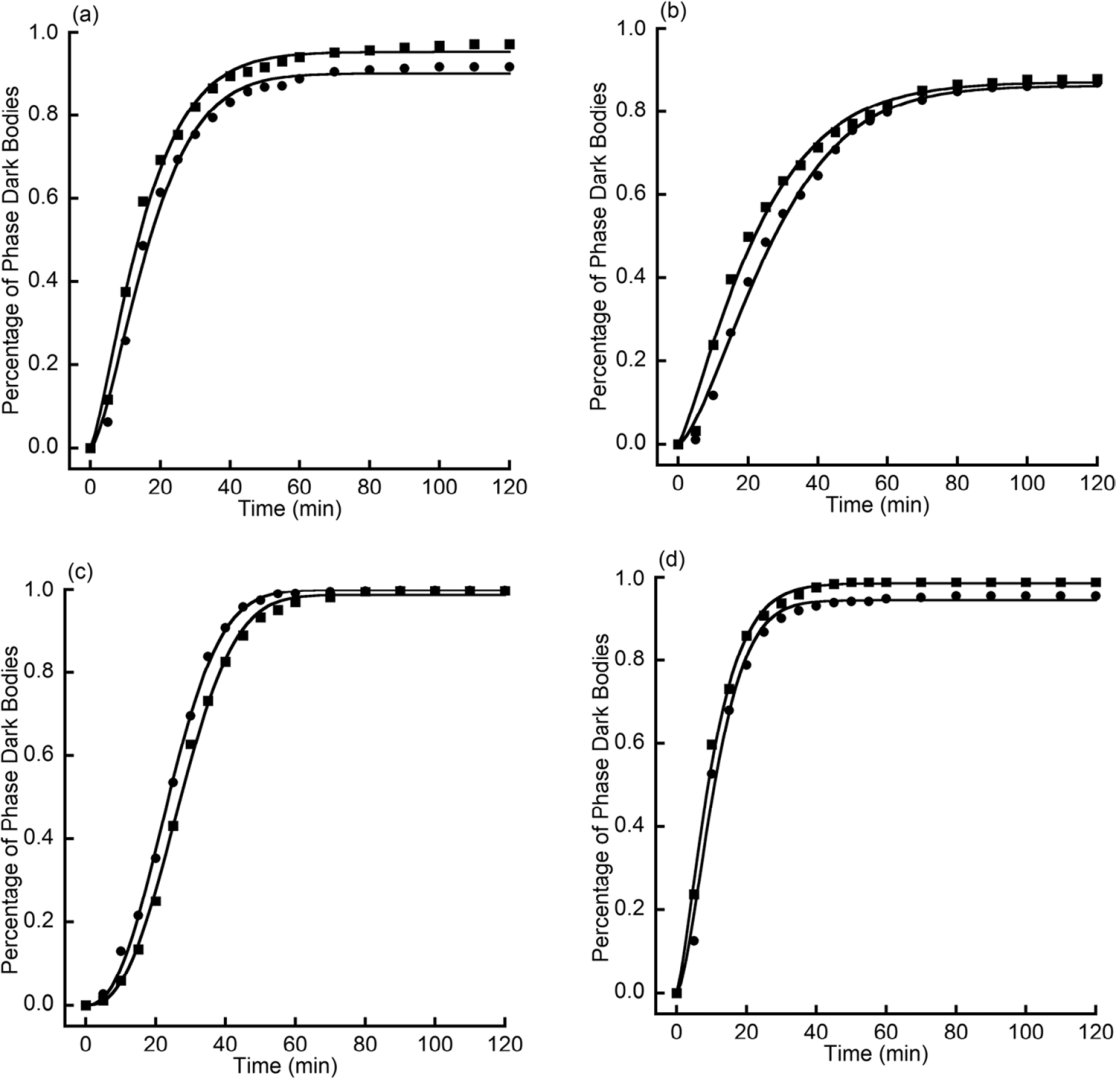
The graphs show the kinetics of germination of multiple individual spores. *B. atrophaeus* spores were germinated with L-alanine (**a**) or AGFK (**b**) or Ca-DPA (**c**) or TSB (**d**) in oxic (●) or anoxic (■) conditions (see Materials and methods). Germination was monitored by phase-contrast microscopy. Data were fitted using Eq. (5)

**Fig. 6:**
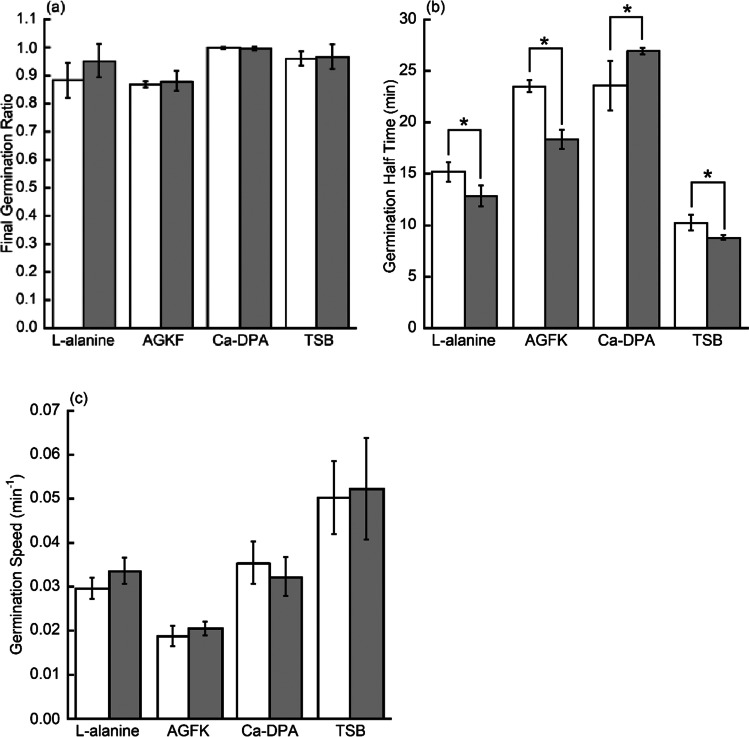
The graphs show the comparison of various germination parameters. *B. atrophaeus* spores were germinated in the oxic (□) or anoxic (■) conditions. Germination was monitored by phase-contrast microscopy. Germination parameters were final germination level (**a**), germination half time (**b**), and germination speed (**c**). Significant differences (*) in germination half time (${t}_{1/2}$) were observed

Curve fitting (${R}^{2}>0.99$ for all 8 fittings) of the average value of triplicate ${R}_{\mathrm{anoxic}}(t)$ and ${R}_{\mathrm{oxic}}(t)$ (Fig. 5) showed similar kinetics with that of ${I}_{\mathrm{anoxic}\_\mathrm{adj}}\left(t\right)$ and ${I}_{\mathrm{oxic}}\left(t\right)$ in L-alanine and AGFK germination. The results (Fig. 6) indicated that in the absence of oxygen, germination half time decreased by 15.3% in L-alanine germination, by 22.1% in AGFK germination, and by 14.0% in TSB germination; however, oxygen had no significant effect on final germination level and germination speed in any of the four cases ($P>0.05$).

**Fig. 7:**
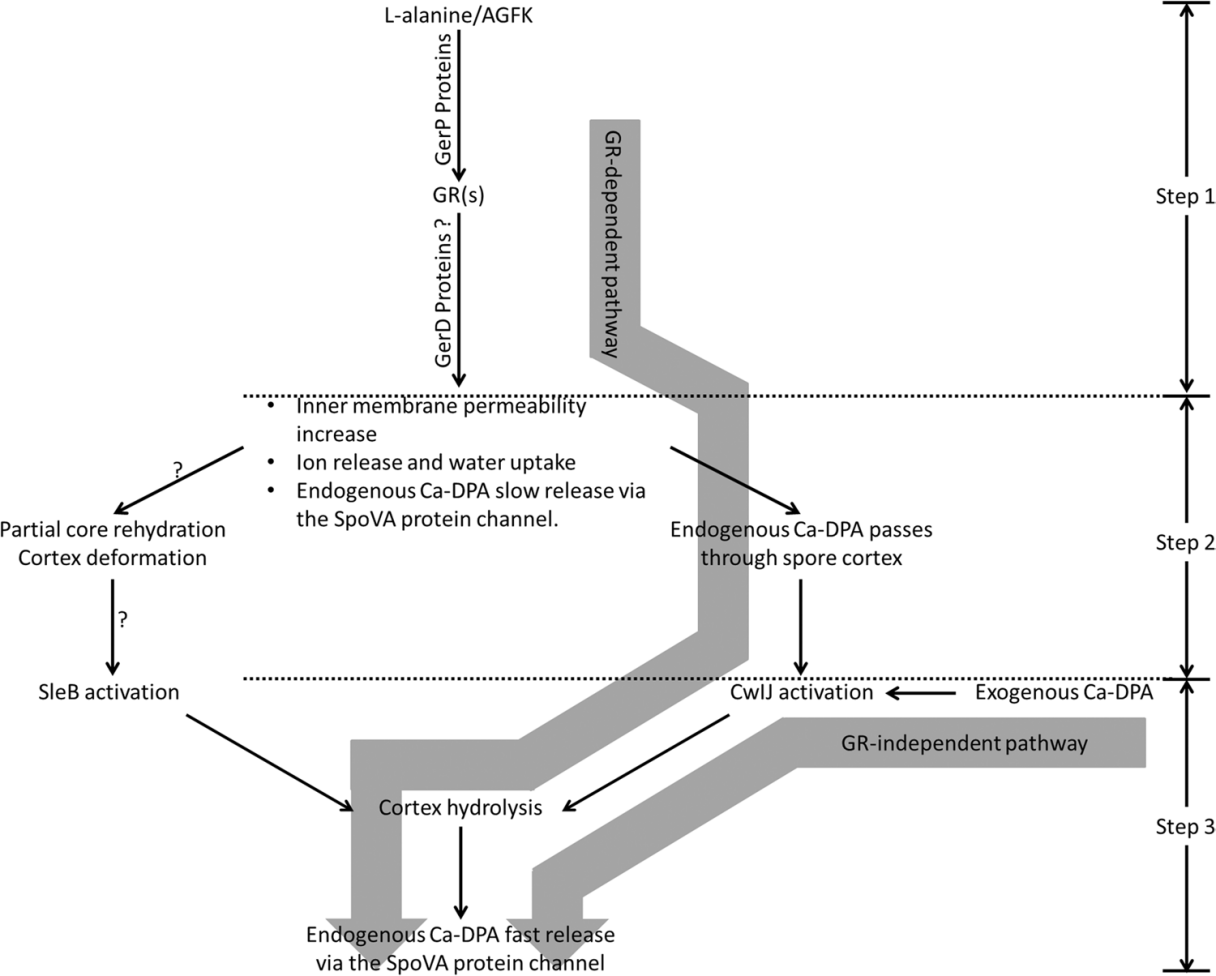
The schematic shows the events in GR-dependent (L-alanine or AGFK induced) or independent (Ca-DPA induced) germination. They have common step 3, while GR-dependent germination has distinct step 1 and step 2

All of the presented results corroborated previous research indicating that aerobic spores may germinate in the absence of oxygen. Additionally, our data demonstrated that germination was facilitated in the lack of oxygen, which has not been previously documented. Germination can be initiated in the laboratory via a GR-dependent or independent pathway, where the biophysical events are partially overlapped (Fig. 7) (Christie et al. 2020; Paredes-Sabja et al. 2014; Setlow 2013, 2014). Our findings indicate that oxygen has a negative effect on only GR-dependent germination, implying that at least one step between steps 1 and 2 (Fig. 7) is oxygen-sensitive.

The primary event in step 1 is the binding of germinant(s) with specific GR(s). GR cooperation is also involved in this process (Atluri et al. 2006). However, it is more plausible that the binding and cooperation are biophysical processes and unaffected by oxygen (Setlow 2003). Apart from GR, two key non-GR proteins are GerP and GerD. GerP proteins can facilitate the movement of some germinants across spore’s outer layers to obtain GR(s) access. However, oxygen is not predicted to alter this movement since the absence of one or more GerP slows both GR-dependent and independent germination (Setlow 2013). GerD is another critical germination protein. GerD deficiency greatly retards GR-dependent germination but has no effect on GR-independent germination (Pelczar et al. 2007), indicating that GerD may act as an intermediary in the processing of signals from GR(s) and determining germination speed (Yi et al. 2010). However, information of the precise locations and the exact functions of GerD remains ambiguous. We hypothesize that GerD-mediated germination signal processing may be oxygen-sensitive.

SpoVA is a critical protein involved in the release of Ca-DPA in step 2. At least a portion of SpoVA composes the channels for Ca-DPA release (Li et al. 2012; Vepachedu et al. 2007). However, there is no indication that the initial slow Ca-DPA release (step 2) and the consequent fast Ca-DPA release (step 3) employ distinct release channels. If this is the case, oxygen should have no effect on SpoVA functions.

Another intriguing subject is the kinetics of the increasing in IM permeability and the opening of the SpoVA channel during Ca-DPA germination. These two events are triggered in some way during GR-dependent germination by internal germination signal(s) from the spore core. However, the recognized role of exogenous Ca-DPA in Ca-DPA germination is to directly activate CwIJ, which then degrades spore cortex (Zhang et al. 2012). Under this condition, it is unclear why IM permeability increases and how the SpoVA channel opens. Is it possible that they are the outcome of the same internal germination signal(s)? If this is the case, where do the germination signal(s) originate? Is it generated when exogenous Ca-DPA interacts with other non-GR proteins (such as GerD)? Due to the fact that events during this time period remains unknown, we are unable to propose any other proteins that may have been implicated at this point.

### Effect of oxygen availability during germination on the culturability of germinated spores

**Fig. 8:**
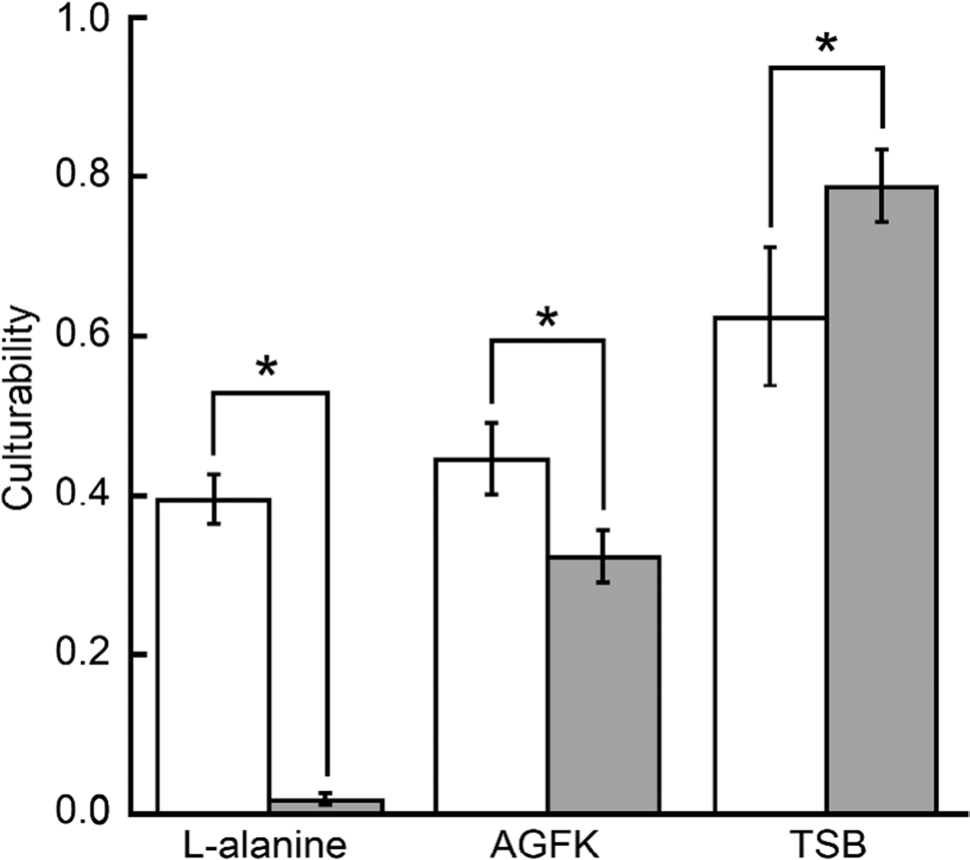
The graph shows the comparison of the culturability of germinated spores germinated in oxic (□) or anoxic (■) conditions. Significant differences (*) in culturability were observed

The culturability ratio ${R}_{i.j}$ was defined as the number of CFU divided by the total number of spores being cultured. Here, *i* stood for oxygen condition (*i* = oxic/anoxic) and *j* stood for germinant (*j* = L-alanine/AGFK/TSB). Results (Fig. 8) indicated that ${R}_{\mathrm{oxic},\mathrm{ L}-\mathrm{alanine}}$ was 20.6 times of ${R}_{\mathrm{anoxic},\mathrm{ L}-\mathrm{alanine}}$ and ${R}_{\mathrm{oxic},\mathrm{ AGFK}}$ was 1.38 times of ${R}_{\mathrm{anoxic},\mathrm{AGFK}}$, while ${R}_{\mathrm{oxic},\mathrm{ TSB}}$ was 79.2% of ${R}_{\mathrm{anoxic},\mathrm{ TSB}}$. Meanwhile, the culturability of germinated spores that germinated by any of the three germinates with or without oxygen did not change significantly over time, at least within 5 h (data not shown), indicating that the time at which germinated spores were transferred to TSA is not critical in this study. The CFU count of culturable bacteria in the anaerobic chamber air was zero, demonstrating that operation without a biosafety cabinet did not invalidate our results. Oxically cultured spores developed CFU within 24 h, but anoxically cultured spores created no detectable CFU after 72 h, indicating that our *B. atrophaeus* is obligate aerobic.

The culturability of germinated spores does not receive enough attention in the field of bacterial spore physiology, most likely because germinated spores are in a special state that is unlikely to exist in nature (the nature environment is either so harsh that spores are kept dormant or rich enough to support spores’ germination followed by outgrowth and vegetative metabolism). We hypothesize that the variation in culturability of germinated spores is more likely connected to events during the germination phase. Based on the energy source of ATP generation, Setlow et al. split the ATP level during germination into three stages (Setlow et al. 1970a). ATP production in stage II is oxygen-dependent (Hyatt et al. 1964), as evidenced by the reduced ATP level of spore germinating in N_2_ (Setlow et al. 1970a). One hypothesis is that in the lack of oxygen, the quantity of ATP was decreased, resulting in incomplete DNA dissociation from α/β-type SASP or inadequate RNA/protein synthesis. In dominant spore, DNA binds with a group of α/β-type SASPs, which at least partially contributes spore’s resistance to wet heat, oxidative agents, and UV radiation. In the SASP-DNA complex, DNA directly contacts with the carboxyl-terminal region of α/β-type SASP (Rao et al. 1992) to form chemical bonds, probably hydrogen bonds (Dev et al. 1990). During germination, DNA dissociates from α/β-type SASP, which is finally degraded to amino acids by sequence-specific protease (Setlow 1988). This dissociation step is critical for spore outgrowth (Hayes et al. 2001). Breaking the chemical bonds between DNA and α/β-type SASP requires energy, and it is possible that the poorer culturability was caused by a lesser quantity of SASP-free DNA owing to a lack of ATP.

Aside from DNA dissociation, ATP levels also influence RNA production. After the onset of *Bacillus megaterium* germination, the turnover stage of RNA synthesis is from 0 to 15 min, while time range of ATP production in stage II is approximately from 5 to 15 min (Setlow et al. 1970b). The overlap showed that inadequate RNA synthesis owing to a shortage of energy might result in an irreversible drop in the culturability of germinated spore.

Protein synthesis is similarly affected by ATP levels. In the first 75 min of *B. megaterium* germination, protease cleaved α/β-type SASPs that dissociated from DNA to small amino acids (Setlow 1988), which is a major protein synthesis source (Setlow et al. 1975). However, *gpr* spores (spores with an inactivated *gpr* gene that were unable to degrade free α/β-type SASPs) exhibited the same final viability as *gpr*^+^ spores (spores with a normal *gpr* gene), despite their slower outgrowth (Sanchez-Salas et al. 1992). As a result, limited protein synthesis is unlikely to be cause of poorer culturability.

## Conclusions and future work

To the best of our knowledge, this is the first report comparing the germination of *B. atrophaeus* spores in oxic and anoxic conditions. Under anoxic conditions, we discover that (1) germination was accelerated in L-alanine, AGFK, and TSB germination but not in Ca-DPA germination; (2) final germination level was not impacted; and (3) germinated spores showed poorer culturablilty even when oxygen was re-supplied during incubation. It is hypothesized that in the absence of necessary oxygen, the biophysical events of germination can still occur at varying rates. However, the biochemical process, presumably DNA-SASP complex dissociation and/or RNA synthesis, will be impeded, resulting in a decreased culturability of germinated spore. Regrettably, comparative studies and literatures are pretty sparse and out of date. Future research should focus on determining which biomolecule(s) are influenced by oxygen. Screening additional species of spore formers, particularly obligate and facultative anaerobes, may also be required to get a more comprehensive conclusion on this issue.

## Data Availability

Yes.
